# The regulation of trophoblast invasion and decidual reaction by matrix metalloproteinase‐2, metalloproteinase‐7, and metalloproteinase‐9 expressions in the rat endometrium

**DOI:** 10.1002/rmb2.12342

**Published:** 2020-08-06

**Authors:** Rasim Hamutoğlu, Hüseyin Eray Bulut, Celal Kaloğlu, Ozan Önder, Tuğba Dağdeviren, Merve Nur Aydemir, Ertan Mahir Korkmaz

**Affiliations:** ^1^ Department of Histology and Embryology Faculty of Medicine Cumhuriyet University Sivas Turkey; ^2^ Cumhuriyet University Assisted Reproduction Technology (ART) Center Sivas Turkey; ^3^ Department of Molecular Biology and Genetics Faculty of Science Cumhuriyet University Sivas Turkey

**Keywords:** decidualization, implantation, MMP, Real‐time PCR, trophoblast invasion

## Abstract

**Purpose:**

We aimed to evaluate how matrix metalloproteinases (MMPs) regulate the trophoblast invasion and placentation.

**Methods:**

Female rats were divided into the estrous cycle and early pregnancy day groups. Obtained uterine tissues and implantation sites were processed for immunofluorescence and real‐time PCR examinations.

**Results:**

The mRNA expression of MMP‐7 was higher than MMP‐2 and MMP‐9. Immunofluorescence findings confirmed that MMP‐2, MMP‐7, and MMP‐9 were localized in the endometrial stroma, while MMP‐7 was high in glandular and lining epithelial cells throughout the entire estrous cycle. However, their immunolocalizations and mRNA expressions were dramatically changed with the early pregnancy days. The MMP‐7 reached very strong immunostaining in the giant trophoblast cells (GTCs), and the cytoplasm of mature and differentiating decidual cells, whereas MMP‐2 and MMP‐9 were mostly seen in the primary decidual zone (PDZ), GTCs, and the endothelium of blood vessels.

**Conclusions:**

All three MMPs seemed likely to be a key mediator of trophoblast invasion into the decidual region as well as angiogenesis during the placentation process. Due to the strong and wide expression of MMP‐7 in the mature decidua, it could be suggested that MMP‐7 is important for decidual ECM remodeling and it might be used as a new marker of decidual reaction.

## 1 INTRODUCTION

Extracellular matrix (ECM) specifically undergoes degradation during the tissue repair and remodeling, morphogenesis, and various signaling activities through its unique biochemical and biomechanical properties.^1^, ^2^

Up to date, nearly about 200 Zn‐dependent matrix metalloproteinases (MMPs), a disintegrin and metalloproteinase with thrombospondin motifs (ADAMTS), and BMP/tolloid proteases found in mammals that function in the ECM metabolism, and contain a large number of endopeptidases, each containing an active site Zn^2+^ ion. Some BMP/tolloid and ADAMTS proteases are required not only for the ECM turnover but also for its assembly through molecular activation or maturation of matrix precursor proteins as well.^3^ MMPs are responsible for the ECM degradation ^4^ and are highly regulated to maintain tissue‐specific activity and a variety of physiological processes.^5^, ^6^ These proteases are related to the decomposition of the ECM components to produce a variety of cellular environments for the execution of fully coordinated mechanisms.^7^, ^8^ MMP activation occurs through proteolytic cleavage or by modifying the thiol group by oxidation. MMPs are regulated by the growth factors (TGF‐β and IGF), cytokines, and angiogenic factors (endothelin‐1) during the implantation process,^9^, ^10^ at different stages ranging from the transcriptional level to activation inhibition of other ECM components.^11^, ^12^ Uncontrolled activity of proteases may lead to various defects such as arthritis, cancer, chronic tissue ulcer, fibrosis, aneurysms, nephritis, encephalomyelitis, and atherosclerosis.^13^, ^14^, ^15^, ^16^, ^17^

In addition to the re‐arrangement of the extracellular structure is critical for the uterine physiology, ECM remodeling plays also a vital role in the successful pregnancy by regulating decidualization ^18^ trophoblast invasion, placental development,^19^, ^20^ and spiral artery regeneration (especially MMP‐2 and MMP‐9).^21^, ^22^ It has been reported that the proliferation and differentiation of uterine stromal cells during decidualization are partially regulated by MMPs and tissue inhibitors of metalloproteinases (TIMPs).^18^ The TIMPs differ in their selectivity for different MMPs.^23^, ^24^ Although TIMP‐1 and TIMP‐2 inhibit the active forms of all MMPs, they are linked to both active and latent forms of MMP‐9 and MMP‐2, which are involved in the breakdown of collagen IV that is the main component of the maternal basal membrane, and are considered key enzymes during implantation. The end of tissue breakdown and bleeding are initiated by the increase in expression of TIMPs, mainly TIMP‐1 and TIMP‐2.^25^ TIMPs produced by trophoblastic and decidual tissues during pregnancy have also an inhibitory effect on activated MMPs, thus limits excessive trophoblast invasion. Uterine trophoblasts and vascular cells are the major sources of MMPs.^26^, ^27^ MMP‐2 and MMP‐9 play a role in the endometrial tissue remodeling during the estrous and menstrual cycles and during pregnancy.^28^, ^29^

Most MMPs are expressed in the human endometrium ^30^ and are present in large amounts during the menstruation and blastocyst implantation.^31^ While the menstrual cycle in humans lasts 28 days, the estrous cycle takes only five days in rats. Therefore to work with rats in such studies is easy to conduct and it was shown that trophoblast invasion, placentation, and decidualization mechanisms are highly similar both in rats and in humans. Moreover, in addition to limited studies and the specific roles of MMP‐2, MMP‐7, and MMP‐9 proteins are not fully highlighted, we think that most of the data obtained from in vitro models are insufficient to understand the mechanism of implantation biology since many critical factors may not be supplied in in vitro cultured systems. In this study, we employed immunofluorescence (IF) staining and real‐time PCR methods to give a more comprehensive tissue function of these proteins during the estrous cycle and early days of pregnancy in the rat endometrium. We showed here that all three MMPs might be crucial for controlling trophoblast invasion and placental development, while only MMP‐7 has a role of decidual cell differentiation and maintain proper/permanent mature decidual matrix. According to our knowledge, this study could be the first to demonstrate the presence of MMP‐7 expression in mature and differentiating decidual cells during implantation, and thus, it might be predicted as a new marker for decidual reaction.

## 2 MATERIALS AND METHODS

### 2.1 Animals

For the present study, 21 adult female *Wistar albino* rats (6‐8 weeks old) weighing between 220 and 250 grams were obtained from the Animal Laboratory of Cumhuriyet University (Sivas, Turkey). The rats were maintained in a temperature‐controlled room (23 ± 2°C) at 60‐70% relative humidity with a 12‐L:12‐D photoperiod cycle. They were fed with standard pellet feed and tap water ad libitum throughout the experiment. Of the total, twelve animals were used for the estrous cycle days (three for each stage), whereas the remaining nine animals were divided into three groups of three for pregnancy days (7.5, 8.5, and 9.5). The status of the estrous cycle, that is, the proestrus, estrus, metaestrus, and diestrus stages, was determined in the non‐pregnant rats by the vaginal smear method.^32^ The rats were mated with fertile males of the same strain overnight in standard plastic cages in the Animal Laboratory of Cumhuriyet University, Sivas, Turkey, to establish a pregnancy. The next morning, mating was confirmed through the presence of a vaginal plug, and the males were prevented from further contact with such females. The sighting of a vaginal plug was declared as Day 1 of pregnancy (1.0 day post‐coium, dpc). All females were sacrificed by rapid decapitation in the adult period. The pregnant females were laparotomized on days 7.5, 8.5, and 9.5 to obtain implantation sites. Uterine horns were dissected. All efforts were made to minimize the number of animals used and their suffering.

The experimental protocols were approved by the Animal Ethics Committee of Sivas‐Cumhuriyet University (approval No: 2017.02.02) and were in compliance with Directive 2010/ 63/EU on the protection of animals for experimental and other scientific purposes, complying ethical standards of the Low animal welfare No 41/2009 as national guides on the care and use of laboratory animals.

### 2.2 Tissue processing

The tissues were fixed in 4% PBS‐buffered paraformaldehyde overnight at 4°C. After dehydration, the tissues were embedded in paraffin, sectioned (thickness, 2 μm), dewaxed in xylene, and stained using hematoxylin & eosin and immunofluorescence staining methods. However, we did not include hematoxylin‐eosin photographs in this article. Serial sections were made.

### 2.3 Histological examination

#### 2.3.1 Light microscopy

Paraffin sections, 2 μm thick, were taken by a rotary microtome (Leica RM 2125RT, Germany). Sections were used for indirect immunofluorescence labeling for MMP‐2, MMP‐7, and MMP‐9 expressions.

#### 2.3.2 Immunofluorescence labeling

Following deparaffinization in xylene and rehydration in decreasing concentrations of ethanol, the sections were washed in distilled water. Antigen retrieval was applied to sections at 95°C, 550W in 10 mmol/L citrate buffer at pH:6 for 5 minutes in a microwave oven. The sections were washed once in phosphate‐buffered saline (PBS)‐Triton‐X 100. In order to prevent non‐specific staining, the sections were detained for 20 minutes in SuperBlock (Sky Tech Lab, USA), followed by incubation of sections in monoclonal mouse anti‐human MMP‐2, MMP‐7, and MMP‐9 primary antibodies (Santa Cruz Biotechnology) overnight at 1:100 dilution. From this point on, all steps were done in dark conditions. The sections were washed in PBS‐Triton‐X, three changes for 5 min, and then incubated for 30 min in goat anti‐mouse immunoglobulin‐G (IgG) secondary antibody (Abcam, USA), which was diluted 1:500 in an antibody diluent reagent (Invitrogen, USA) at pH: 7.4. They were washed again in PBS‐Triton‐X with three changes, and nuclear staining was carried out in 4'6'‐diamidine‐2‐phenyl‐indole dihydrochloride (DAPI) (200 nm/mL) for 5 min. Following three washes in PBS‐Triton‐X 100, the sections were evaluated using a fluorescence microscope (Olympus BX51). All steps in the immunostaining process were applied to the negative control sections without the primary antibody incubation. Photographs from the convenient fields of view were taken using an Olympus BX51 (Tokyo, Japan), and a semi‐quantitative scoring method was applied.

### 2.4 Quantification of mRNA expression

**FIGURE 1:**
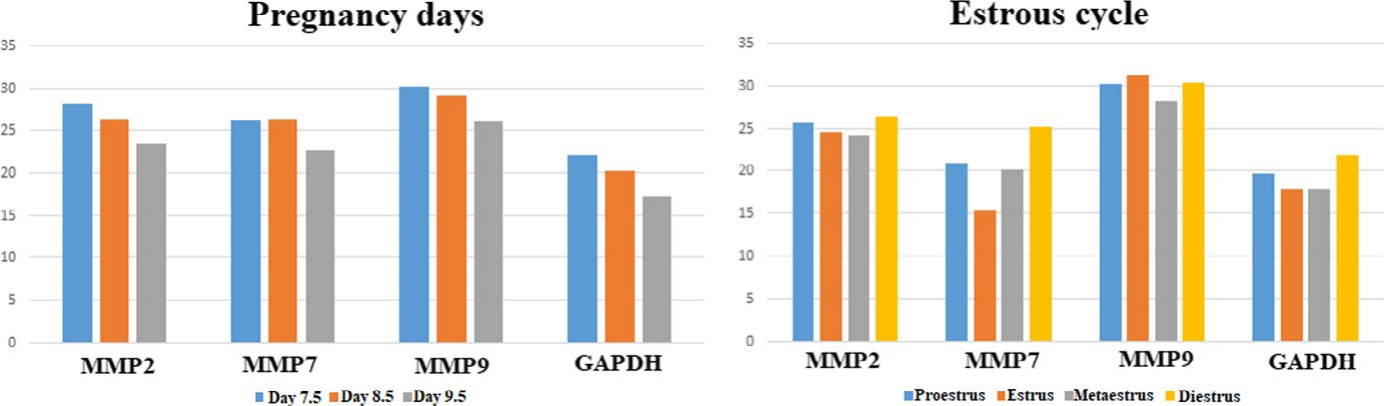
*Ct* values of MMP‐2, MMP‐7, and MMP‐9 mRNAs for estrous cycle and pregnancy days

**FIGURE 2:**
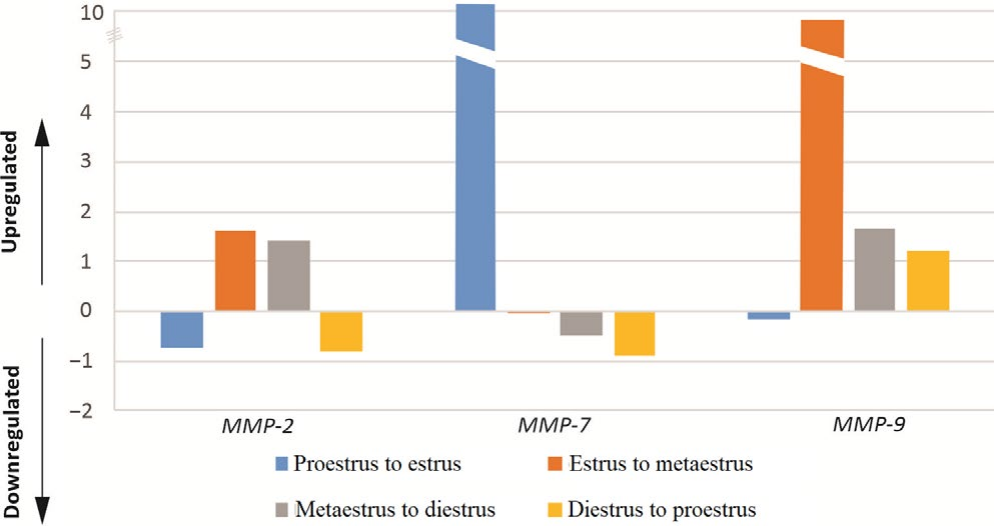
Relative expression ratios of MMP genes showing relative changes in the estrous cycle stages (n = 3) after normalization to GAPDH. It has been determined that MMP‐2 gene was expressed at decreasing levels in transition from the proestrus to estrus and the diestrus to proestrus, and it was expressed in increasing levels in the transition from the estrus to metaestrus and metaestrus to diestrus. The MMP‐7 gene was expressed as upregulated in the transition from the proestrus to estrus, while the MMP‐9 gene was downregulated. Therefore, it can be said that MMP‐7 and MMP‐9 genes display inverse‐related expression patterns

**FIGURE 3:**
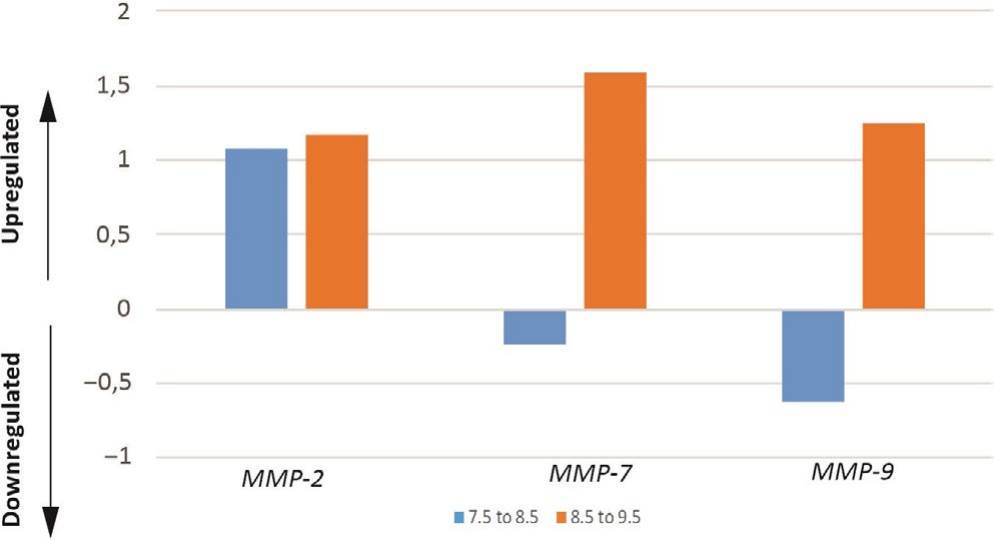
Relative expression ratios of MMP genes showing relative changes in the early pregnancy days (n = 3) after normalization to GAPDH. It was observed that MMP‐2 had an increased level of expression in the transition from pregnancy days of 7.5‐8.5 and 8.5‐9.5. MMP‐7 and MMP‐9 genes were downregulated in the transition from the pregnancy days of 7.5‐8.5, while they were upregulated in the transition from the days of 8.5‐9.5

The uterine tissues were removed from the non‐pregnant and pregnant rats, followed by euthanasia, and tissue was obtained by scraping the uterine epithelium through curettage. The scraped samples were placed in RNA later, which is an aqueous, non‐toxic solution that rapidly permeates tissue to stabilize and protect the cellular RNA content,^33^ and the samples were stored at − 20°C. The quantity and quality of total RNA were determined by spectrometry and denaturing agarose gel electrophoresis, respectively. Total RNA was obtained using the RNA isolation kit (RNeasy Mini Kit, Invitrogen) to determine the expression of MMP‐2, MMP‐7, and MMP‐9 mRNAs. cDNAs were synthesized from the RNAs obtained from these mRNAs using the cDNA kit (Superscript II‐RT, Thermo Fisher Scientific), and the expression of these mRNAs was determined by real‐time PCR using relative quantitation. PCR amplification was performed using Sso Advanced Universal SYBR Green Supermix compatible with Bio‐Rad CFX Connect. Primers were designed to amplify cDNAs of around 100 bp to maximize efficiency and are summarized in Table 1. Own designed primers were used in the present study. All cDNA concentrations were set to 100 ng/μl. RNA integrity was verified by agarose gel electrophoresis/ethidium bromide staining and by OD_260/280_ absorption ratio. Polymerase chain reaction cycle parameters were 95°C for 15 sec and 60°C for 1 min for 40 cycles. The threshold line was set in the linear region of the plots above the baseline noise, and threshold cycle (Ct) values were determined as the cycle number at which the threshold line crossed the amplification curve. No primer‐dimer formation was observed during the 40 PCR amplification cycle. Polymerase chain reaction without template or template substituted with total RNA was used as a negative control to verify experimental results. After amplification, the specificity of the PCR was confirmed by melt curve analysis. Each quantitation was reproduced three times, and each quantified gene has the Ct normalized against the *GAPDH* gene, constitutively expressed reference gene. Reference *GAPDH* and *MMP* genes expression levels in the estrous cycle and early pregnancy days were given in Figure 1. A value of 2^ΔΔCt^ (ΔΔCt=(Ct_target gene1_ ‐ Ct_reference gene(_ *_GAPDH_*_)_) _estrous cycles/early pregnancy days_ ‐ (Ct_target gene2_ ‐Ct_reference gene(_ *_GAPDH_*_)_) _estrous cycles/early pregnancy days_) was calculated over Ct values and normalized to the selected reference genes. The aim is to determine the increase or decrease in the relative quantification of expression levels of genes. Relative expression levels which calculated from the formula of 2^−ΔΔCt^ of the estrous cycle and the early pregnancy days were given in Figure 2 and Figure 3, respectively.

**TABLE 1:** Primers and their accession numbers used in real‐time PCR

Primers	Sequence (5’→3’)	Product size (bp)	Tm value (°C)	T_A_ value (°C)	GenBank accession nos.	Product location
*MMP‐2* Forward	CAGGGAATGAGTACTGGGTCTATT	24	63.6	56.7	NM_031054	1904‐1927
*MMP‐2* Reverse	ACTCCAGTTAAAGGCAGCGTCTAC	24	65.2	56.7	NM_031054	1999‐2022
*MMP‐7* Forward	ACTCATGAACTTGGCCACTCTC	22	62.1	58	NM_012864	665‐686
*MMP‐7* Reverse	TTTCCATATAACTTCTGGATGCCT	24	60.3	58	NM_012864	784‐807
*MMP‐9* Forward	ATCTCTTCTAGAGACTAGGAAGGAG	25	64.1	58	NM_031055	2221‐2245
*MMP‐9* Reverse	CAAGCTGATTGGTTCGAGTAGC	22	62.1	58	NM_031055	2329‐2350
*GAPDH* Forward	CTCTCTGCTCCTCCCTGTTC	20	62.5	58	NM_017008.4	1‐20
*GAPDH* Reverse	GCCAAATCCGTTCACACCG	19	59.5	58	NM_017008.4	87‐105

## 3 RESULTS

The immunolocalizations and expressions of MMP‐2, MMP‐7, and MMP‐9 were determined using immunofluorescence and real‐time PCR methods, and the results were presented in tables (Tables 2 and 3) and figures (Figures 2 and 3). All sections were examined by two independent observers, and evaluations were made in the uterine tissue based on the degree of staining of these proteins.

**TABLE 2:** MMP‐2, MMP‐7, and MMP‐9 immunolocalizations during the estrous cycle

Proteins	Endometrial sites/Estrous cycle	Proestrus	Estrus	Metaestrus	Diestrus
MMP‐2	Luminal epithelium	−	+	+	++
	Glandular epithelium	−	−	+	++
	Subepithelial stroma	+	+++	++	++
	Basal stroma	+++	++++	+++	+++
	Blood vessels	+++	+++	+	+++
MMP‐7	Luminal epithelium	+++	++++	+	+++
	Glandular epithelium	++	++++	−	++++
	Subepithelial stroma	+	−	++	−
	Basal stroma	+	+	++++	+
	Blood vessels	++++	+++	++	+
MMP‐9	Luminal epithelium	+	+	+	+
	Glandular epithelium	−	+	+	+
	Subepithelial stroma	++	++	+	+++
	Basal stroma	+++	++++	++	++++
	Blood vessels	+++	++++	+	+++

Note

++++, very strong; +++, strong; ++, moderate; +, weak; − negative.

**TABLE 3:** MMP‐2, MMP‐7, and MMP‐9 immunolocalizations during early pregnancy days

Proteins	Regions	Implantation sites/Pregnancy Days	Day 7.5	Day 8.5	Day 9.5
MMP‐2	Anti‐mesometrial region	Primary decidual zone	++	+++	++
		Secondary decidual zone	+	++	+
		Basal zone	+	+	+
		Embryo site Reichert's Membrane	++	+	+++
			*	++	++
		Giant Trophoblast Cells	*	+++	+++
		Ectoplacental Cone	*	+++	+++
	Mesometrial region	Uterine epithelium	++	*	*
		Blood vessels	+++	+++	+++
		Basal zone	+++	+++	+++
MMP‐7	Anti‐mesometrial region	Primary decidual zone	++	++	+
		Secondary decidual zone	+	+++	++++
		Basal zone	++	++++	++++
		Embryo site	+++	+++	++++
		Reichert's Membrane	*	+++	++++
		Giant Trophoblast Cells	*	+++	++++
		Ectoplacental Cone	*	+++	++++
	Mesometrial region	Uterine epithelium	+	*	*
		Blood vessels	++	+++	+++
		Basal zone	++	+++	+++
MMP‐9	Anti‐mesometrial region	Primary decidual zone	++	+++	+
		Secondary decidual zone	+++	++	+
		Basal zone	++	+	+++
		Embryo site	+	++	++
		Reichert's Membrane	*	++	++
		Giant Trophoblast Cells	*	+++	+++
		Ectoplacental Cone	*	+++	+++
	Mesometrial region	Uterine epithelium	+	*	*
		Blood vessels	+++	+	+++
		Basal zone	++	+	+++

Note

++++, very strong; +++, strong; ++, moderate; +, weak; ‐ negative; *, not observed

### 3.1 Immunofluorescence of MMP‐2, MMP‐7, and MMP‐9 proteins in the estrous cycle

Semi‐quantitative results of the MMPs immunolocalizations during the estrous cycle in rat endometrium are shown in Table 2.

#### 3.1.1 Proestrus

**FIGURE 4:**
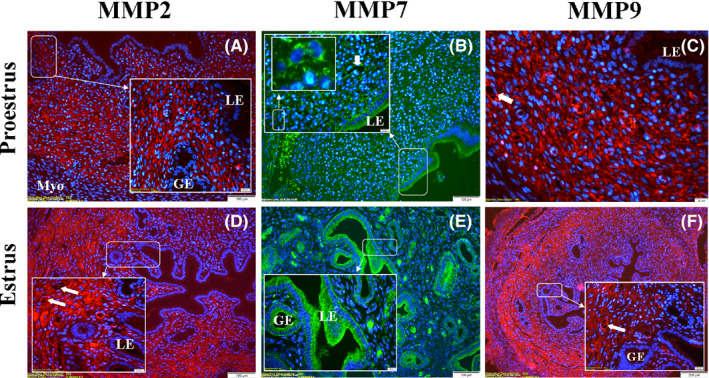
The morphological changes and MMP immunolocalizations in the rat endometrium during proestrus and estrus stages. MMP expressions showed mostly strong immunolocalization in the blood vessels (arrow). MMP‐7 showed very strong immunolocalization in the luminal (LE) and glandular (GE) epithelia in the estrus stage, especially the cytoplasm of stromal cells (b,e). High magnification images showed strong immunolocalization in the blood vessels (arrowheads) (c,d,e), luminal (b,e), and glandular (e) epithelia. Myometrium (Myo)

The immunolocalization of MMP‐7 in the luminal epithelium was strong; however, there was found that no MMP‐2 and MMP‐9 immunostainings in the glandular epithelium. (Figure 4A–C). Strong/very strong immunostaining was observed in all MMPs in the endothelium of the blood vessel. MMP‐2 and MMP‐9 immunolocalizations, except for MMP‐7, increased gradually from the subepithelial stroma to the basal stroma (Figure 4A and C). There was found that very strong MMP‐7 immunolocalization in the cytoplasm of stromal cells rather than the stromal region in the endometrium (Figure 4B) (Table 2).

#### 3.1.2 Estrus

The immunolocalization of MMP‐7 in the luminal and glandular epithelia was very strong (Figure 4E). MMP‐2 and MMP‐9 immunolocalizations, except MMP‐7, increased gradually from subepithelial stroma to basal stroma as in the proestrus stage, and there was also found that strong MMP‐7 immunostaining in the cytoplasm of stromal cells rather than the stromal region (Figure 4E). MMP‐2 and MMP‐7 showed high levels of immunolocalization in the endothelium of the blood vessels, but MMP‐9 was stronger (Figure 4F–h) (Table 2).

#### 3.1.3 Metaestrus

**FIGURE 5:**
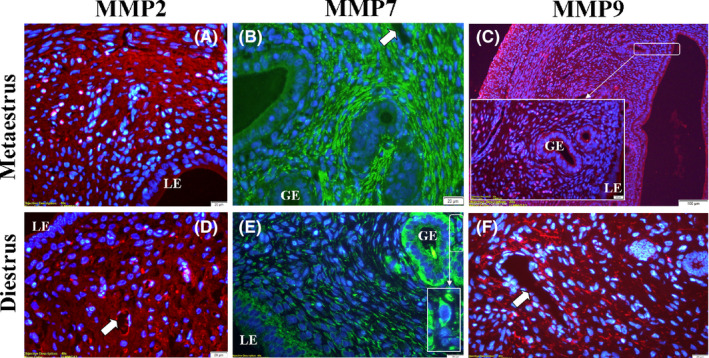
The morphological changes and MMP immunolocalizations in the rat endometrium during metaestrus and diestrus stages. MMP‐2 and MMP‐9 expressions showed mostly strong immunolocalization in the stromal region of endometrium (a,c,d,f). MMP‐7 showed very strong immunolocalization in the luminal (LE) and glandular (GE) epithelia in the diestrus stage and especially cytoplasm of stromal cells (e‐high magnification image) just as proestrus and estrus stages (b,e). Blood vessels (arrow)

Immunolocalization levels of all MMPs in luminal and glandular epithelia were quite low (Figure 5A–C). Increasing and strong immunolocalization levels of all MMPs from the subepithelial stroma to the basal stroma were observed, but this was slightly weaker in MMP‐9 (Figure 5C) (Table 2).

#### 3.1.4 Diestrus

MMP‐7 immunolocalization was detected as strong and very strong in the luminal and glandular epithelia, respectively (Figure 5E). MMP‐7 expression levels were also detected very strong in stromal cells located in the endometrial stromal region as in the proestrus and estrus stages (Figure 5E). MMP‐2 and MMP‐9 immunolocalization levels were found to be strong in the endothelial part of the blood vessels, whereas MMP‐7 was relatively weak (Figure 5D and F) (Table 2).

### 3.2 Expression levels of MMPs in the estrous cycle

Real‐time PCR was used to evaluate the changes in levels of MMP‐2, MMP‐7, and MMP‐9 transcripts in the estrous group uterine tissues, and normalization was achieved with GAPDH as the reference gene. GAPDH, which is a housekeeping gene, was used to demonstrate the stability of expression level. The *ct* values obtained after real‐time PCR with MMP‐2, MMP‐7, and MMP‐9 primers from cDNA samples taken from the uterine tissues are shown in Figure 1. MMP‐7 gene expression had a higher expression value than MMP‐2 and MMP‐9 in all cycle stages. The mRNA expressions of all three MMPs were reached the lowest level in the diestrus stage (Figure 1). The fold change expression of MMPs was detectable in all of the endometrial tissues investigated. While MMP‐7 gene expression had a higher expression value than MMP‐2 and MMP‐9 in the transition from the proestrus to estrus, it was determined that MMP‐9 gene had a higher expression value than the others in the transition from the estrus to metaestrus, metaestrus to diestrus, and diestrus to proestrus (Figure 2).

Looking at the gene expression of MMP genes several times, the expression level in the estrus stage of MMP‐7 gene increased by 43.11% compared to the proestrus stage. Its expression increased by 25.63% in the transition from the estrus stage to the metaestrus stage. The lowest expression level was observed in the diestrus stage. There was a 30.9‐fold increase in the transition from metaestrus to diestrus stages. It could be said that the expression of MMP‐7 gene expression in the estrous cycle increases to a very high level of expression, especially in the estrus stage (Figure 2).

Expression level of the MMP‐2 gene showed an increase of 2.14% in the estrus stage compared to the proestrus stage, whereas it was observed that an increase of 1.43% in the expression level of the metaestrus stage compared to the estrus stage. The stage of diestrus has the lowest rate of expression of this gene. MMP‐2 gene expression level increased by 5.24% in metaestrus stage compared to diestrus stage, while expression levels of proestrus and estrus stages increased by 1.7% and 3.65%, respectively (Figure 2).

The lowest expression level in MMP‐9 gene expression was observed in the estrus stage. MMP‐9 gene expression levels in the proestrus stage increased by 2.07% compared to the estrus stage. MMP‐9 gene expression level in metaestrus stage increased by 8.16% and 4.46%, respectively, when compared to estrus and diestrus stages (Figure 2).

### 3.3 MMP expressions in early pregnancy

#### 3.3.1 Day 7.5 of pregnancy

**FIGURE 6:**
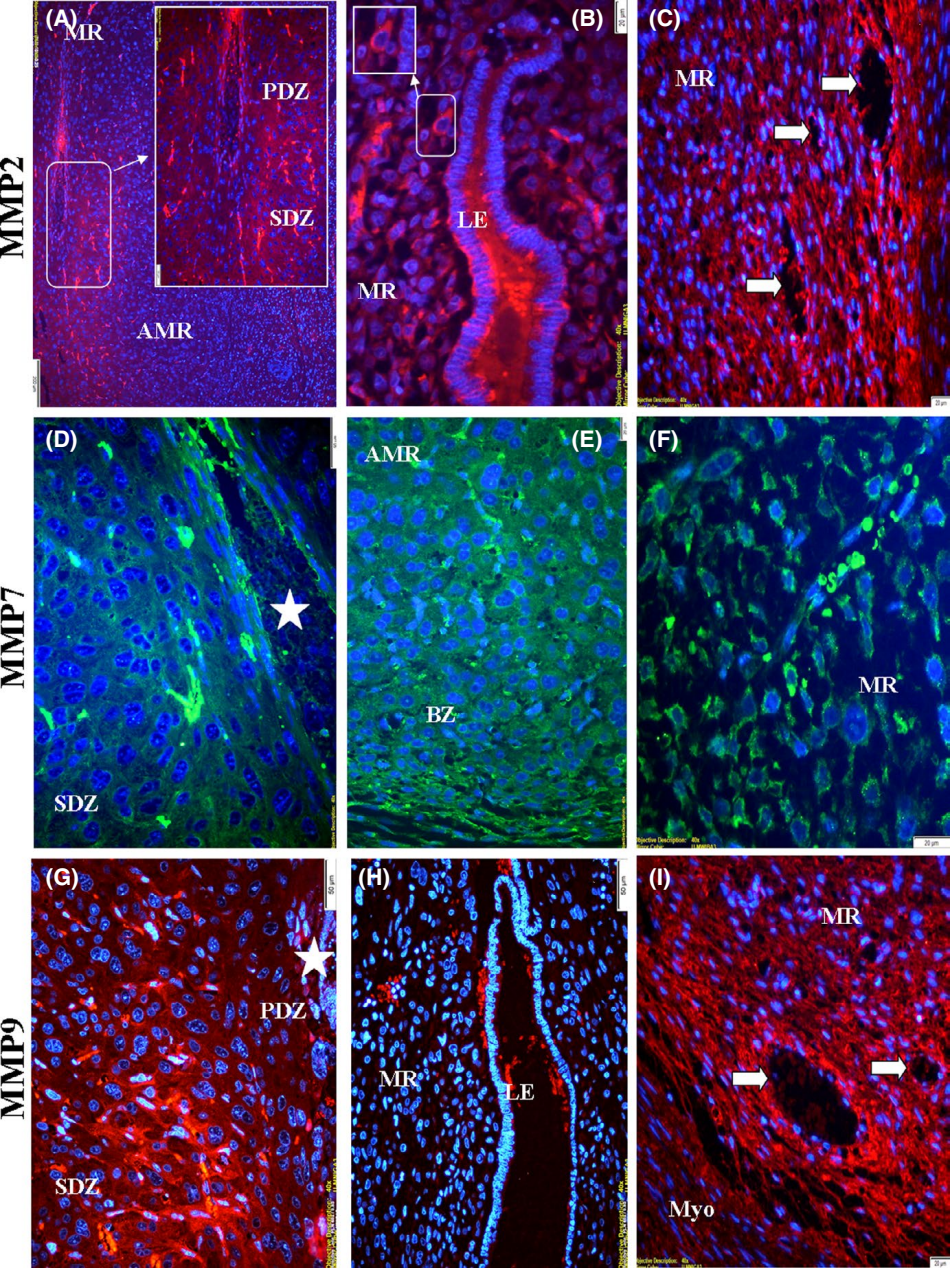
The immunolocalization of MMPs in the rat endometrium during pregnancy day of 7.5. High magnification image showed strong immunolocalization in the cytoplasm of undifferentiated stromal cells(b). Primary decidual zone (PDZ), secondary decidual zone (SDZ), mesometrial region (MR), embryo (asterisk), blood vessels (arrow), anti‐mesometrial region (AMR), luminal epithelium (LE), basal zone (BZ), myometrium (Myo)

Immunolocalization levels of all three proteins were observed to be less strong in the PDZ surrounding the embryo site (Figure 6A,D,g and h). Immunolocalization levels of MMP‐2 and MMP‐9 increased in SDZ than PDZ (Figure 6A, g and h); however, the level of MMP‐7 was the opposite in these decidual regions (Figure 6D and E). The level of MMP‐7 immunolocalization in the cytoplasm of stromal/undifferentiated decidual cells in the mesometrial basal zone was quite strong (Figure 6F). The immunostaining level of MMP‐7 was found to be less strong than other proteins in the endothelial part surrounding the lumen of blood vessels (Figure 6C,i) (Table 3).

#### 3.3.2 Day 8.5 of pregnancy

**FIGURE 7:**
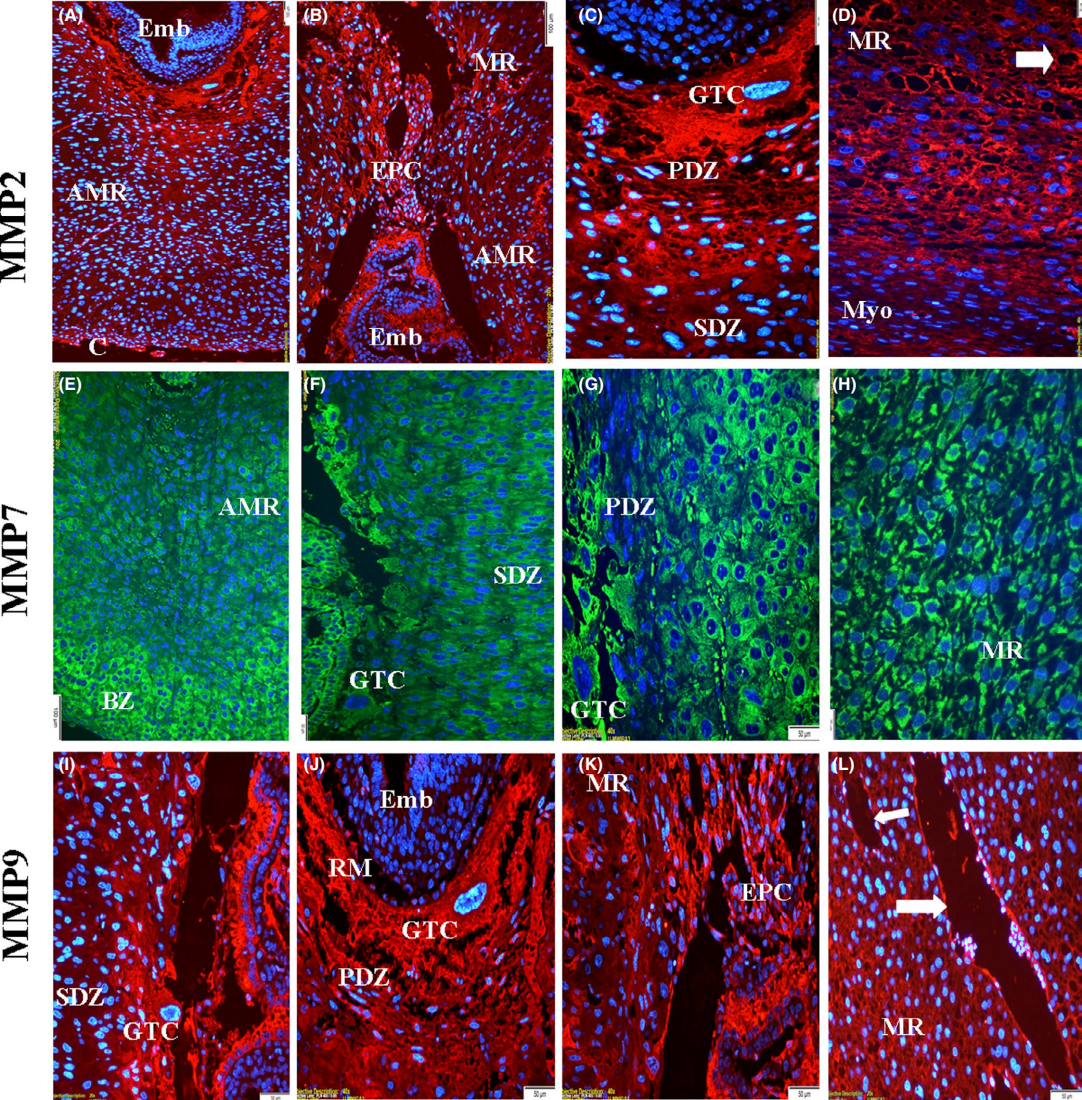
The immunolocalization of MMPs in the rat endometrium during pregnancy day of 8.5. MMP‐2 and MMP‐9 showed strong immunolocalizations in the giant trophoblast cells (GTCs) and primary decidual zone (PDZ), while MMP‐7 showed strong/very strong immunolocalizations in the secondary decidual zone (SDZ), basal zone (BZ) of anti‐mesometrial region (AMR), cytoplasm of undifferentiated stromal cells, and mature decidual cells. Reichert's membrane (RM), mesometrial region (MR), embryo (Emb), blood vessels (arrow), capsule (C), ectoplacental cone (EPC), myometrium (Myo)

Immunolocalization of MMP‐7 was stronger in the embryonal region compared to other proteins (Figure 7B,F and j). Immunostaining of MMP‐2 and MMP‐9 was found to be strong in PDZ (Figure 7C,i,j). The immunolocalization of all three proteins in GTC regions was determined as strong (Figure 7B,C,g and j). Immunostaining of MMP‐2 and MMP‐9 proteins was determined to be strong in the endothelial part of the blood vessels (Figure 7D and l). The expression levels of MMP‐7 protein in the cytoplasm of decidual cells in the anti‐mesometrial region (especially basal zone), cells in the embryo site (Figure 7A and B), and undifferentiated stromal cells in the mesometrial region (Figure 7H) were quite strong during this period of pregnancy (Table 3).

#### 3.3.3 Day 9.5 of pregnancy

**FIGURE 8:**
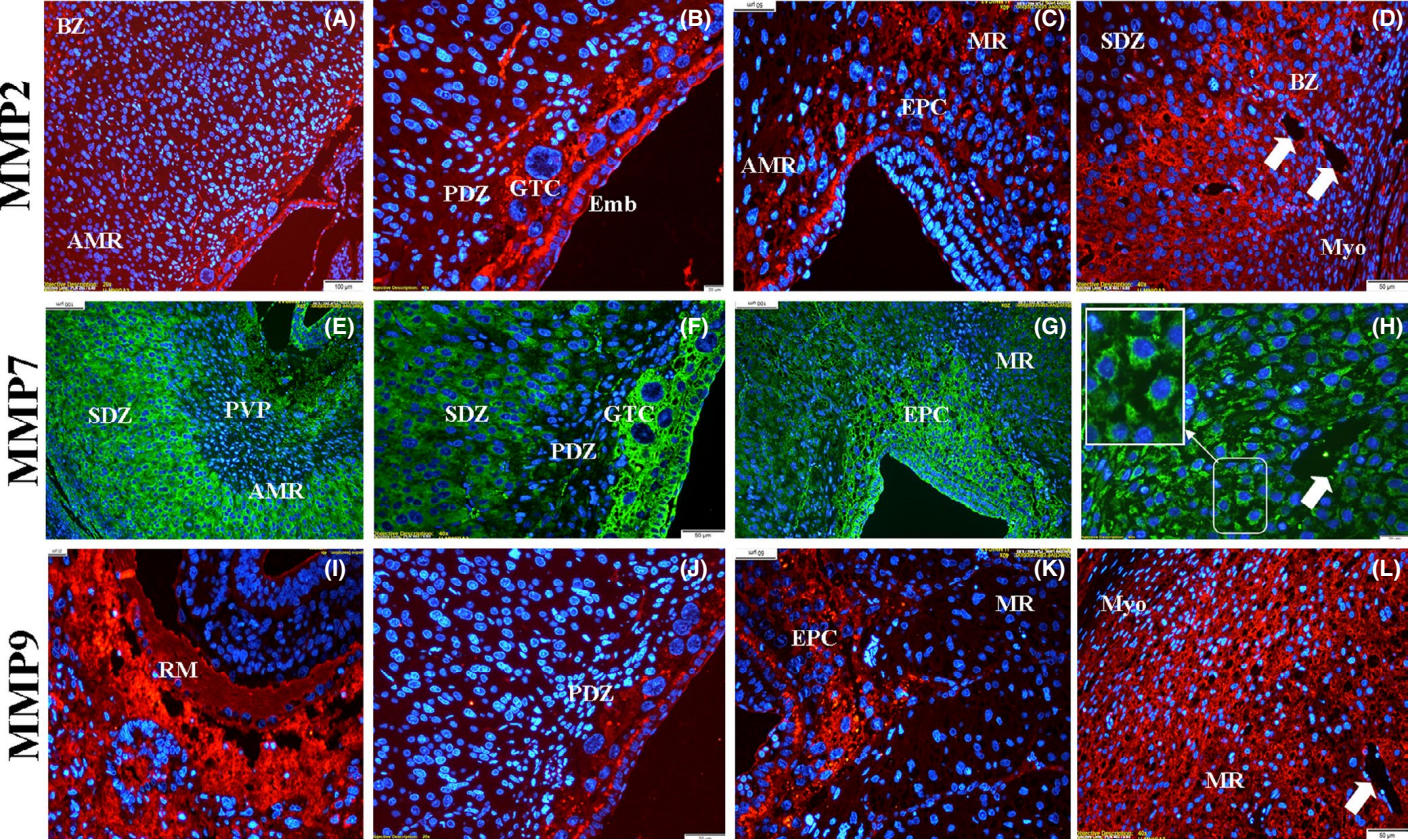
The immunolocalization of MMPs in the rat endometrium during pregnancy day 9.5. MMP‐7 showed very strong immunolocalization in the giant trophoblast cells (GTCs), secondary decidual zone (SDZ), and basal zone (BZ) of anti‐mesometrial region (AMR), cytoplasm of undifferentiated stromal cells (h‐high magnification image), and mature decidual cells. Primary decidual zone (PDZ), primitive vitellus sac placenta (PVP), mesometrial region (MR), myometrium (Myo), embryo (Emb), blood vessels (arrow), ectoplacental cone (EPC), Reichert's membrane (RM)

It could be easily said that MMP‐7 exhibited a tremendously strong immunolocalization, mainly in the cytoplasm of decidual cells located in the anti‐mesometrial and mesometrial regions, GTCs, and EPC, as well as the embryo region, compared to other MMPs (Figure 8E‐h). The immunolocalization level of MMP‐2 and MMP‐9 was particularly strong in GTCs (Figure 8A,B and j). Immunolocalization of all three proteins was found to be strong in the endothelial part of the blood vessels in this region (Figure 8D,h and l) (Table 3).

### 3.4 Expression levels of MMP genes in early pregnancy

MMPs genes were highly induced by day 9.5 of pregnancy, but especially the level of MMP‐2 and MMP‐7 mRNAs was higher than those of MMP‐9 during days 7.5‐9.5 of pregnancy. It was determined that MMP‐7 reached the highest level when three genes were compared to each other (Figure 1).

Figure 3 shows the real‐time PCR results of MMP expression quantification values on pregnancy days. It was observed that MMP‐2 exhibited a relative increase, MMP‐7 and MMP‐9 genes were downregulated in the transition from the pregnancy days of 7.5‐8.5, and upregulated in the transition from the pregnancy days of 8.5‐9.5. In total, MMP‐7 and MMP‐9 were determined to be more expressed than MMP‐2 between 7.5 and 9.5 days (Figure 3). It was determined that MMP‐2 had an increased level of expression in the transition from pregnancy days of 7.5‐8.5 and 8.5‐9.5.

## 4 DISCUSSION

MMPs are responsible for the tissue degradation and turnover of ECM components in several physiological processes.^34^ Bearing in mind that MMPs had a central role in endometrial ECM remodeling, it has been shown in the present that MMP‐2, MMP‐7, and MMP‐9 proteins were expressed at relatively constant values in the endometrial stroma and MMP‐7 in glandular and lining epithelial cells at high levels throughout the entire estrous cycle, but their expression was dramatically changed with the early pregnancy days.

During the estrous cycle, consistent with previous reports,^35^, ^36^, ^37^ our immunofluorescence results confirmed that all three MMPs could involve in the tissue remodeling, probably under the influence of estrogen and progesterone. In addition, it seems that MMP‐7, which is an epithelium‐specific protein (Figure 5E), is more effective than other MMPs in the regulation of the luminal re‐epithelialization and gland formation. The real‐time PCR analysis of the present study revealed that MMP‐7 mRNA reached a very strong expression level, especially in the estrus stage. On the other hand, decreased expression levels of all MMPs in diestrus stage may result from an increase in progesterone activity. Consistent with the data of the present study, Feng et al ^38^ found that the highest levels of uterine tissue concentration of MMP‐7 during the proestrus and estrus stages were higher than those found in metestrus and diestrus stages. Slayden and Brenner ^39^ also stated that progesterone plays an intricate role by suppressing the transcription of MMPs such as MMP‐1, MMP‐3, MMP‐7, MMP‐8, and MMP‐10 by inhibiting the expression of MMP‐inducing factors (such as cytokines). They also expressed that it could play a complex role in the secretory phase by moderately inhibiting MMP‐2 and MMP‐9 expressions in cultured cells. In addition to MMP gene regulation, it should be noted that the expressed various growth factors and cytokines in response to ovarian steroids might affect MMP expression in the endometrium.

The main objective of the present work was to elucidate whether the localization and mRNA expression of these proteins change with decidualization and trophoblast invasion, which are very critical for a successful and ongoing pregnancy. Endometrial stromal cells transform into decidual cells that have epithelial characteristics and produce many factors including growth factors, cytokines, and basal lamina‐like ECM.^40^ Herein, we observed that MMP‐7 was specifically expressed in mature (Figure 7E‐g) and differentiating decidual cells (Figure 8H); however, MMP‐2 and MMP‐9 had an intensive expression in blood vessels and undifferentiated stromal cells in the basal zone. Thus, our results confirm that while MMP‐7 is an epithelial‐specific MMP as mentioned above, on the other hand, allow us to suggest that MMP‐7 should be considered as a marker of the decidual reaction. Our real‐time PCR analysis also confirmed that all three MMPs were expressed at increasing levels in the rat endometrium. The different localization and expression of MMP‐7 compared to MMP‐2 and MMP‐9 during the estrous cycle and early pregnancy days may depend on various mechanisms that regulate these proteases. In addition well‐known factors affecting MMP expression,^41^ recent reports have mentioned that miRNAs can also interestingly regulate MMP secretion in the decidua. For example, Sirohi et al ^42^ hypothesized that miR‐145 may be involved in the expression of MMP‐9 in endometrial stromal cells. They found that MMP‐9 was suppressed in decidual cells having overexpression of miR‐145. In addition, Su et al ^22^ indicated that the activity and expression of MMP‐2 and MMP‐9 were also downregulated by miR‐346 and miR‐582‐3p, which in turn compromised the ability of trophoblast cell invasion.

Although there are many factors secreted from decidual tissue that may regulate the trophoblast invasion, it seems that MMP‐TIMP interaction is also a very critical event at the embryo‐uterine interface.^43^ For example, conditioned medium from in vitro decidualized stromal cells (DCM) has a strong stimulatory effect on the invasion of ACH3P, JEG‐3, and HTR8/SVneo cells. DCM increases the expression of MMP‐2, MMP‐3, and MMP‐9, and inhibits the expression of TIMP‐1, TIMP‐2, and TIMP‐3 in ACH3P cells. DCM increased the expression of TIMP‐2 in JEG‐3 cells and repressed TIMP‐1 and TIMP‐3 in the trophoblast cells.^44^ Meanwhile, TIMP‐1 forms a stable complex with MMP‐9 to block the activation of MMP‐9 thereby indirectly inhibit excessive invasion of trophoblast cells.^45^, ^46^, ^47^ Maintaining a dynamic balance of MMP‐9/TIMP‐1 is suitable for embryo implantation, can prevent damage to endometrial tissue with an excessive invasion of trophoblast cells, and can smoothen the pregnancy process.^47^, ^48^ Thus, an altered ratio of MMPs and TIMPs, along with activation of other proteases, would allow the degradation of the extracellular matrix for the optimal trophoblast invasion.^43^ At weeks 6‐8 of a human pregnancy, high expression of MMP‐2 (at later decreasing concentrations) dominated the condition on MMP‐9, whereas MMP‐9 expression increased between 8th and 11th weeks, predominating until the end of pregnancy,^49^ which led to the conclusion that MMP‐2 plays a vital role during implantation and MMP‐9 plays an essential role during invasion.^50^ The expression of MMP‐9 by trophoblast cells, as we also showed here, which possesses basement membrane‐degrading proteolytic activity, is required to facilitate successful implantation and endometrial invasion.^49^ Moreover, in recent reports, through a reduced hatching rate and MMP‐9 expression in* *in vitro culture condition has been linked to low implantation rate.^51^, ^52^, ^53^

In rodents, the primary decidual zone consisted of the closely packed decidual cells with tight junctions, which has been considered as a barrier zone against the trophoblasts.^54^ Moreover, since this area has collapsed blood vessels, this barrier should be removed gradually for the trophoblasts to reach the blood vessels and to form yolk sac placenta and chorioallantoic placenta at the anti‐mesometrial and mesometrial pole, respectively.^55^ As observed in the present study, although all three MMPs had strong expression in GTCs and ectoplacental cone, MMP‐2 and MMP‐9 proteins had much higher immunolocalization in the primary decidual zone and endothelial cells of blood vessels (Figure 7A‐C,i,j), while MMP‐7 staining was negative in the primary decidual zone (Figure 8E,F). Based on these results, our previous report showed that laminin and fibronectin expression was diminished in the primary decidual zone.^20^ Therefore, MMP‐2 and MMP‐9 may directly participate in ECM degradation by trophoblast invasion, but MMP‐7 may regulate this indirectly at the cellular level.

Numerous studies have indicated that matrix metalloproteinases (MMPs), such as MMP‐2, MMP‐7, MMP‐9, and TIMP‐1, which mediate the process of extracellular matrix (ECM) remodeling, play a critical role in cell migration progress, and are a prerequisite for angiogenesis.^56^, ^57^, ^58^, ^59^, ^60^ Reynolds and Redmer ^61^ indicated that the formation of new blood vessels is requisite for placentation and MMPs can contribute to angiogenesis. Besides, Kraling et al ^62^ stated that the balance between MMPs and their inhibitors must be restored when newly formed blood vessels have matured to support basement membrane and endothelial cell differentiation, and MMPs promote endothelial cell migration and tube formation by proteolytically remodeling the basement membrane.^63^, ^64^ MMP‐2 and MMP‐9 are particularly emphasized since type IV collagenase activities are very important in the first stage of angiogenesis.^65^ Sun et al ^66^ also found that MMP‐2 might play a more important role than MMP‐9 in platelet microvesicle‐enhanced angiogenesis. Wu et al ^67^ stated that the ERK and AKT signaling pathways are known to be involved in the expression of MMPs associated with angiogenesis. In our study, we clearly showed that all three MMPs had strong immunofluorescence signals in EPC, and MMP‐2 and MMP‐9 showed intense staining in the endothelium of maternal blood vessels of the mesometrial decidua in which chorioallantoic placenta forms. Based on these results, it should be considered that MMPs have critical roles during placental angiogenesis, and alterations in the MMPs expressions could be involved in infertility and pregnancy complications ^50^, ^68^ in addition to endometrial disorders.^69^, ^70^, ^71^, ^72^

In conclusion, considering the importance of MMPs for invasion and remodeling processes, our results may provide that the temporal regulation of all three MMPs may be crucial for controlling trophoblast invasion and placental development, while MMP‐7 has a role of decidual cell differentiation and may be predicted a new marker for decidual reaction because it is wide distribution through the mature decidua during the peri‐implantation period. Further studies are needed to identify the molecular signals involved in the regulation of expression and activity of studied MMPs.

## DISCLOSURES

*Conflicts of Interest:* The authors declare that there is no conflict of interest that could be perceived as prejudicing the impartiality of the research reported.

*Human and animal rights:* This article does not describe any experiments involving human participants. All of the institutional and national guidelines for the care and use of laboratory animals were followed. The protocol for the research project was approved by a suitably constituted ethics committee.
